# The cell cloud: Adopting systems biology concepts in the era of single-cell immunology

**DOI:** 10.1371/journal.pbio.3003853

**Published:** 2026-07-02

**Authors:** Tal Shay, Christophe O. Benoist, Ricardo Grieshaber-Bouyer

**Affiliations:** 1 Department of Life Sciences, Ben-Gurion University of the Negev, Beer Sheva, Israel; 2 Immunological Genome Project Consortium; 3 Department of Immunology, Harvard Medical School, Boston, Massachusetts, United States of America; 4 Deutsches Zentrum Immuntherapie, Erlangen, Germany; 5 Department of Internal Medicine 3 – Rheumatology and Immunology, Friedrich-Alexander-Universität (FAU) Erlangen-Nürnberg and Universitätsklinikum Erlangen, Erlangen, Germany

## Abstract

Immune cells exist as continuous clouds, not discrete categories. In this Perspective, authors from the Immunological Genome Project reframe immune identity through systems biology, and redirect where immunotherapies should aim: at the dynamics of the cloud, not just its center.

## Teaser

High-throughput single-cell assays reveal data that defies discrete categorization. The ‘cell cloud’ model, grounded in established systems biology principles, offers a framework to navigate biological plasticity alongside technical variability.

**Fig 1:**
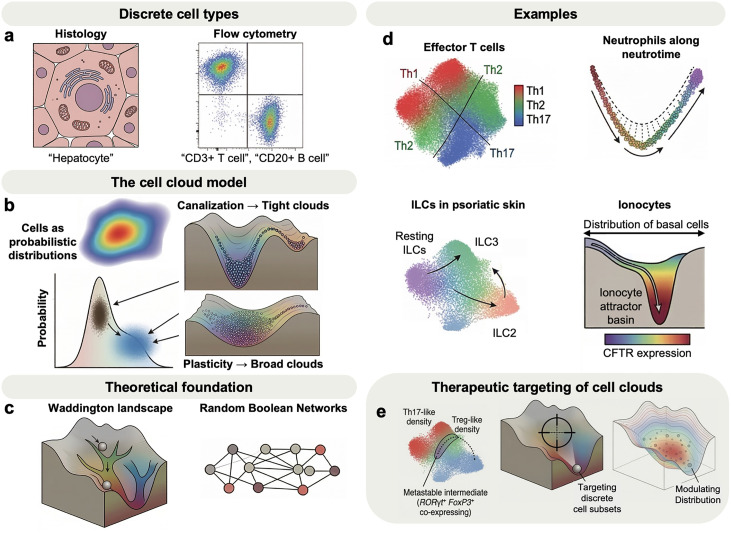
The cell cloud model: Theory, examples, and therapeutic implications. **(a)** Historically, discrete cell types were defined by histology or standard flow cytometry. **(b)** “Cell clouds” represent cells as the probability distribution of transcriptional states they occupy. Cell clouds serve as the empirical manifestation of attractor basins. Canalization produces tighter clouds, and cellular plasticity produces broader clouds. **(c)** Theoretical foundation. Waddington”s epigenetic landscape illustrates differentiation as cells settling into stable attractor basins. Stable states are defined by Random Boolean networks with recurrent, stable configurations of gene expression that are robust to perturbations. **(d)** Empirical observations of cell clouds. **(e)** Therapeutic targeting. Advanced immunotherapies must shift from targeting simplified discrete subsets to modulating the overarching probability distribution of the cell cloud or neutralizing specific metastable intermediates. The panels in this Figure were generated with the assistance of Google Gemini.

For over a century, the “cell type” has served as the atomic unit of multicellular biology. Beginning with histological classifications (i.e., hepatocyte) and later refined by flow cytometry gates (i.e., T cells instead of lymphocytes), immunologists have operated under the paradigm that cells belong to discrete, stable categories defined by distinct surface markers or transcriptional programs (Fig 1a). Within cell types, phenotype can be transiently modified by spatial context, cell cycle, metabolite availability, circadian changes, etc., collectively termed cell “states” [1]. The cell type, overlaid by cell state, has been enormously productive for decades. However, the advent of single-cell RNA sequencing (scRNA-seq) and related high-throughput “omics” methods, which allow profiling of thousands of features across millions of cells, has revealed a continuous, probabilistic landscape of cellular identities, in which some regions have high occupancy probability, representing traditional cell types, and some regions have low occupancy probability, representing, for example, transition states (Fig 1b). This continuity cannot be attributed to technical limitations alone, as it reflects a biological truth that systems biologists have modeled for decades, and immunologists now have to acknowledge and integrate.

The probabilistic nature of cellular identity has a rich theoretical foundation. Waddington’s epigenetic landscape framed differentiation as a ball rolling down valleys, each representing a stable basin of attraction for a particular cell state. Each cell within this basin was considered as belonging to this cell type with bulk measurements, and the size and steepness of the basin reflect the effort needed to transition from this cell type to another one (Fig 1c) [2]. The mathematical framework underlying this landscape was formalized by Kauffman’s random Boolean networks, in which cell types correspond to attractor states: recurrent, stable configurations of gene expression that are robust to perturbations [3]. In this view, the gene regulatory network (GRN) of an immune lineage may anchor the cellular identity via quasi-potentials, exemplified by toggle switches within a GRN whose two stable states correspond to two cell types. Such a toggle switch is the GATA1/PU.1 circuit governing hematopoietic commitment, where high GATA1 represses PU.1 to drive erythropoiesis, while high PU.1 represses GATA1 thus driving myelopoiesis. Notably, in the “view from the nucleus” GRN, the GRN includes only the active interactions in the specific cell at the specific time (in contrast to the “view from the genome” GRN, which is static and identical in all cells of the same organism) [4]. Cells within a lineage share the same GRN that defines and stabilizes the basin of attraction those cells occupy in the gene expression space.

A cell cloud is the empirical manifestation of an attractor basin: the collection of transcriptional states a cell probabilistically occupies while fluctuating within its lineage (Fig 1b). The breadth of a cloud reflects the steepness of the Waddington valley. Deep, steep valleys produce tight, narrow clouds (canalization); shallow valleys allow broad, diffuse clouds (plasticity). Continuous “smears” between clouds correspond to cells traversing ridges between adjacent attractor basins, a phenomenon now central to trajectory inference methods in scRNA-seq [5].

Several recent immunological datasets illustrate this continuum (Fig 1d). Gene expression programs corresponding to “classical” effector T cell populations such as T helper 1 (T_H_1), T_H_2, and T_H_17 are continuously distributed within the lineage; any discrete partition is arbitrary [6]. Similarly, neutrophils in homeostasis are distributed along a single, chronologically ordered trajectory, termed “neutrotime”, that can be sliced into an arbitrary number of “subsets” [7]. In psoriatic skin, innate lymphoid cells (ILCs) do not partition into discrete ILC1–3 groups but form a continuous cloud, with ILC3-like transcriptomes arising from both ILC2-like cells and resting ILCs [8]. In each case, continuity within an attractor basin is the biological signal, not noise.

Importantly, the cloud model does not replace discrete cell types; it complements them. The discovery of the pulmonary ionocyte via scRNA-seq illustrates this clearly: a previously invisible, rare cell type defined by sharply peaked expression of the anion channel CFTR, accompanied by genes associated with ion transport and fluid regulation, that is now implicated in cystic fibrosis pathogenesis [9]. Though located on a continuous developmental trajectory with basal cells, the ionocyte occupies a deep, narrow attractor basin (Fig 1d). Ionocytes were identified precisely because scRNA-seq resolves the full landscape. The cell cloud model thus expands the toolkit for cell-type discovery: cell types are regions of high probability density within a continuous landscape, not exceptions to it.

A critical challenge is distinguishing the true biological structure of the cloud from variability that is due to measurement. The molecular basis of cloud variance in the phenotypic space is probably attributed, at least in part, to variance in transcriptional bursting. Transcription occurs in stochastic pulses rather than continuously, such that two biologically identical cells can display different transcript counts depending on when they were sampled in the burst cycle [10]. This intrinsic biological stochasticity must be distinguished from technical artifacts inherent to scRNA-seq. Dropout (such as via incomplete capture of mRNA, limited reverse transcription, or insufficient sequencing) introduces false zeros that artificially expand cell cloud boundaries and create spurious gradients between unrelated cells. Low sequencing depth increases dropout rate, but as sequencing depth and cell number represent a fundamental design trade-off, atlas-scale experiments prioritize broad sampling to map the full landscape. Thus, atlases are often less accurate for resolving the internal texture of individual clouds (e.g., subtle T cell state gradients), which demands deeper per-cell coverage [5].

The cell cloud model has direct implications for immunology and therapeutics. Defining a cell type by thresholding on levels of a handful of surface markers identifies only the dense region of an attractor. The shape and boundaries of a cloud, and its connectivity to neighboring attractors, require high-dimensional analysis. Co-expression of the transcription factors RORγt and FOXP3 in single cells, for instance, marks metastable intermediates between T_H_17 and regulatory T cell attractor basins: functionally distinct from either stable state, transient due to their inherent instability, and potentially targetable through modulation of the underlying quasi-potential landscape. Immunotherapies targeting specific cell populations need to account not only for the attractor center, but for the full probability distribution of phenotypic states that cells within that cloud can occupy (Fig 1e).

The transition from discrete cell types to probabilistic clouds is not a crisis for immunology but an opportunity. The theoretical and mathematical framework for this transition, developed in systems biology over the past half-century, is mature and now made empirically accessible by single-cell technologies. A cell type is a quasi-discrete attractor: stable, reproducible, and biologically meaningful, yet embedded in a continuous landscape of related states. By adopting this framework, immunologists can break their silo and move from cataloguing immune cell types to understanding the dynamic forces that generate and maintain immune cell identity.

## Abbreviations

GRN: gene regulatory network
ILC: innate lymphoid cell
scRNA-seq: single-cell RNA sequencing

## References

[1] Wagner A, Regev A, Yosef N. Revealing the vectors of cellular identity with single-cell genomics. Nat Biotechnol. 2016;34(11):1145–60. doi: 10.1038/nbt.3711 27824854 PMC5465644

[2] Enver T, Pera M, Peterson C, Andrews PW. Stem cell states, fates, and the rules of attraction. Cell Stem Cell. 2009;4(5):387–97. doi: 10.1016/j.stem.2009.04.011 19427289

[3] Kauffman SA. The origins of order: self-organization and selection in evolution. New York: Oxford University Press; 1993.

[4] Davidson EH, Rast JP, Oliveri P, Ransick A, Calestani C, Yuh C-H, et al. A genomic regulatory network for development. Science. 2002;295(5560):1669–78. doi: 10.1126/science.1069883 11872831

[5] Saelens W, Cannoodt R, Todorov H, Saeys Y. A comparison of single-cell trajectory inference methods. Nat Biotechnol. 2019;37(5):547–54. doi: 10.1038/s41587-019-0071-9 30936559

[6] Kiner E, Willie E, Vijaykumar B, Chowdhary K, Schmutz H, Chandler J, et al. Gut CD4+ T cell phenotypes are a continuum molded by microbes, not by TH archetypes. Nat Immunol. 2021;22(2):216–28. doi: 10.1038/s41590-020-00836-7 33462454 PMC7839314

[7] Grieshaber-Bouyer R, Radtke FA, Cunin P, Stifano G, Levescot A, Vijaykumar B, et al. The neutrotime transcriptional signature defines a single continuum of neutrophils across biological compartments. Nat Commun. 2021;12(1):2856. doi: 10.1038/s41467-021-22973-9 34001893 PMC8129206

[8] Bielecki P, Riesenfeld SJ, Hütter J-C, Torlai Triglia E, Kowalczyk MS, Ricardo-Gonzalez RR, et al. Skin-resident innate lymphoid cells converge on a pathogenic effector state. Nature. 2021;592(7852):128–32. doi: 10.1038/s41586-021-03188-w 33536623 PMC8336632

[9] Plasschaert LW, Žilionis R, Choo-Wing R, Savova V, Knehr J, Roma G, et al. A single-cell atlas of the airway epithelium reveals the CFTR-rich pulmonary ionocyte. Nature. 2018;560(7718):377–81. doi: 10.1038/s41586-018-0394-6 30069046 PMC6108322

[10] Raj A, van Oudenaarden A. Nature, nurture, or chance: stochastic gene expression and its consequences. Cell. 2008;135(2):216–26. doi: 10.1016/j.cell.2008.09.050 18957198 PMC3118044

